# A combination of scanning electron microscopy and broad argon ion beam milling provides intact structure of secondary tissues in woody plants

**DOI:** 10.1038/s41598-022-13122-3

**Published:** 2022-06-01

**Authors:** Tomohiro Hatano, Satoshi Nakaba, Yoshiki Horikawa, Ryo Funada

**Affiliations:** 1 United Graduate School of Agricultural Science, Tokyo University of Agriculture and Technology, Saiwai-cho, Fuchu, Tokyo 183-8509 Japan; 2 JEOL Ltd., Musashino, Akishima, Tokyo 196-8558 Japan; 3 Faculty of Agriculture, Tokyo University of Agriculture and Technology, Saiwai-cho, Fuchu, Tokyo 183-8509 Japan; 4 Institute of Global Innovation Research, Tokyo University of Agriculture and Technology, Harumi-cho, Fuchu, Tokyo 183-8538 Japan

**Keywords:** Biological techniques, Imaging, Microscopy

## Abstract

The secondary tissues of woody plants consist of fragile cells and rigid cell walls. However, the structures are easily damaged during mechanical cross-sectioning for electron microscopy analysis. Broad argon ion beam (BIB) milling is commonly employed for scanning electron microscopy (SEM) of hard materials to generate a large and distortion-free cross-section. However, BIB milling has rarely been used in plant science. In the present study, SEM combined with BIB milling was validated as an accurate tool for structural observation of secondary woody tissues of two samples, living pine (*Pinus densiflora*) and high-density oak wood (*Quercus phillyraeoides*), and compared with classical microtome cross-sectioning. The BIB milling method does not require epoxy resin embedding because of prior chemical fixation and critical point drying of the sample, thus producing a three-dimensional image. The results showed that xylem structures were well-preserved in their natural state in the BIB-milled cross-section compared with the microtome cross-section. The observations using SEM combined with BIB milling were useful for wide-area imaging of both hard and soft plant tissues, which are difficult to observe with transmitted electron microscopy because it is difficult to obtain sections of such tissues, particularly those of fragile reaction woods.

## Introduction

Wood is a major terrestrial carbon biomass^1^ used both as a carbon–neutral material and an energy source. Understanding the intact ultrastructure of the secondary xylem is essential as it relates to wood quality^2,3^. However, woody plants have three-dimensional (3D) heterogeneous and microscopic structures, and the imaging methods for these anatomical structures require high resolution^4^. Transmission electron microscopy (TEM) has higher-resolution imaging performance than other techniques, and it has provided much information on the ultrastructure of the secondary cell wall and its formation process^5–8^. Contrary to the observation of a single histological section using TEM or transmitted light microscopy, scanning electron microscopy (SEM) observation provides high-resolution and stereoscopic information on a large area, facilitating the understanding of the patterns of wood formation and microstructure^9–11^. TEM is not always suited for observing the microfibrils of cell walls because polysaccharides irregularly combine with the heavy metal ions in the stain^12^. For this reason, SEM is often used as an alternative means of examining cellulose microfibrils’ orientation in the cell wall of higher plants^12–14^. However, in microscopic observations of cross-sectional biological tissues, the conventional method of microtome sectioning requires embedding with epoxy resin. Additionally, mechanical sectioning of high-density wood frequently requires previous softening by chemical treatment or boiling^15,16^. Even after this softening process, it is often difficult to produce a high-quality and large area cross-section for microscopic observation^17^. Moreover, in SEM observations, embedding with epoxy resin leads to loss of depth and information.

The ion beam milling method based on sputtering is used in electron microscopy for analyzing hard materials because it can provide stress- and strain-free mechanical cutting planes^18–20^. Moreover, a focused ion beam (FIB) with a gallium ion source allows precise milling. Therefore, tomography with FIB-SEM is used for the 3D reconstruction of cell organelles^21,22^. However, FIB can only process a small area, limiting its use for large samples^23^. Therefore, broad argon ion beam (BIB) milling can be applied to cross-sectional SEM analysis, and it is widely used for sampling hard materials^24,25^. The BIB milling process can be performed on a few millimeters of material and adjusted to the required depth^26^; therefore, it is suitable for preprocessing cross-sections before SEM. In addition, BIB milling produces cross-sections with very low damage compared to FIB milling^27,28^. The setting of specimens on the BIB milling system is very easy, and the cross-section to be processed is positioned protruding from the shield plate several tens of micrometers^28^. The protruded portion is then milled by BIB irradiation. However, BIB milling cannot precisely position the cross-section because the portion protruding from the shield plate has to be manually adjusted. Although BIB milling is appropriate for analyzing SEM images, few applications are available for biological samples^28,29^. To the best of our knowledge, no study has been conducted on SEM observations of secondary tissues in woody plants using the BIB milling technique. This may be because the ion beam irradiation BIB milling can induce heat damage on the processing surface, particularly organic materials^30^.

In the present study, we compared the BIB milling method with the conventional microtome sectioning method and validated the potential of combining SEM with BIB milling to observe the secondary tissue microstructures and reaction wood of two woody plants, *Pinus densiflora* (Japanese red pine) and *Quercus phillyraeoides* (Ubame oak). *P. densiflora* consists of tracheids with thick cell walls and its resin duct system has soft tissues and cavities^31^. *Q. phillyraeoides* has a high-density hard-xylem cells with thick cell walls^32^. In addition, *Q. phillyraeoides* develops a thick gelatinous layer (G-layer) of tension wood that can be easily detached from the secondary walls by mechanical stress; therefore, conducting cross-sectional observations by SEM using a microtome method is difficult. Given the above-mentioned characteristics, Japanese red pine and Ubame oak were selected as suitable materials for validating the effectiveness of using SEM combined with BIB milling to observe the secondary tissue microstructure of woody plants. How to prevent sample heating damage by ion beam irradiation, which is a disadvantage of BIB milling, is also discussed.

## Results

### Xylem to phloem radial cross-section prepared by BIB milling

**Figure 1:**
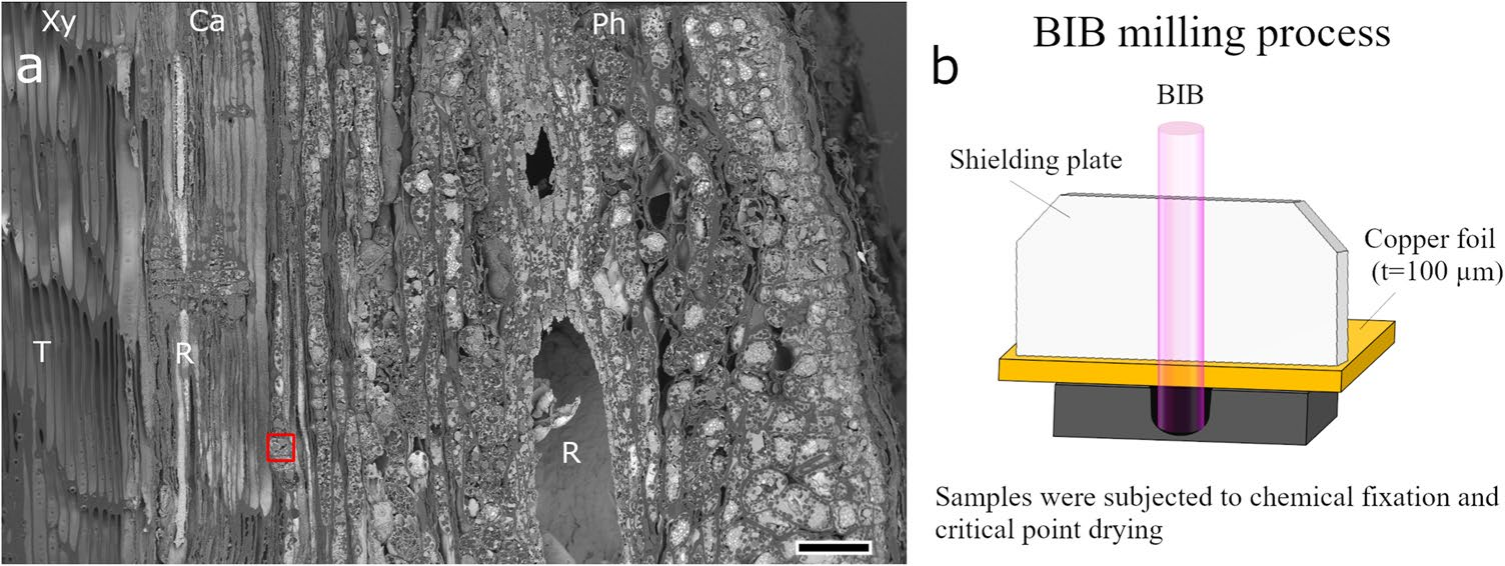
Scanning electron microscopy results of the broad argon ion beam (BIB)-milled radial sections of *Pinus densiflora* (**a**) and illustration of the BIB milling process (**b**). Using the BIB milling process, a broad cross-section from phloem to xylem was obtained without cutting defects and distortions. The resin canal can be easily distinguished from the neighboring tracheids as the resin shows high contrast in the back-scattered electron image. A red-boxed area corresponds to Fig. 2a. Ca, cambium; Ph, phloem; R, resin canal; T, tracheids; Xy, xylem. Scale bar = 100 µm.

Figure 1a shows a BIB-milled radial cross-section of *P. densiflora*. Before BIB milling, we performed chemical fixation and critical point drying of the samples. Figure 1b is a schematic depiction of the BIB milling process. The BIB milling process created a distortion-free cross-section from the xylem to the bark. Because pine resin, rich in unsaturated fatty acids, reacts with osmium tetroxide^33^, the resin canals showed high contrast in the back-scattered electron image. Mechanical cross-sectioning frequently separates the cambium; however, this separation did not occur in the BIB-milled cross-section (Fig. 1a).

### Structure of the radial parenchyma secondary phloem cells prepared by BIB milling and microtome methods

**Figure 2:**
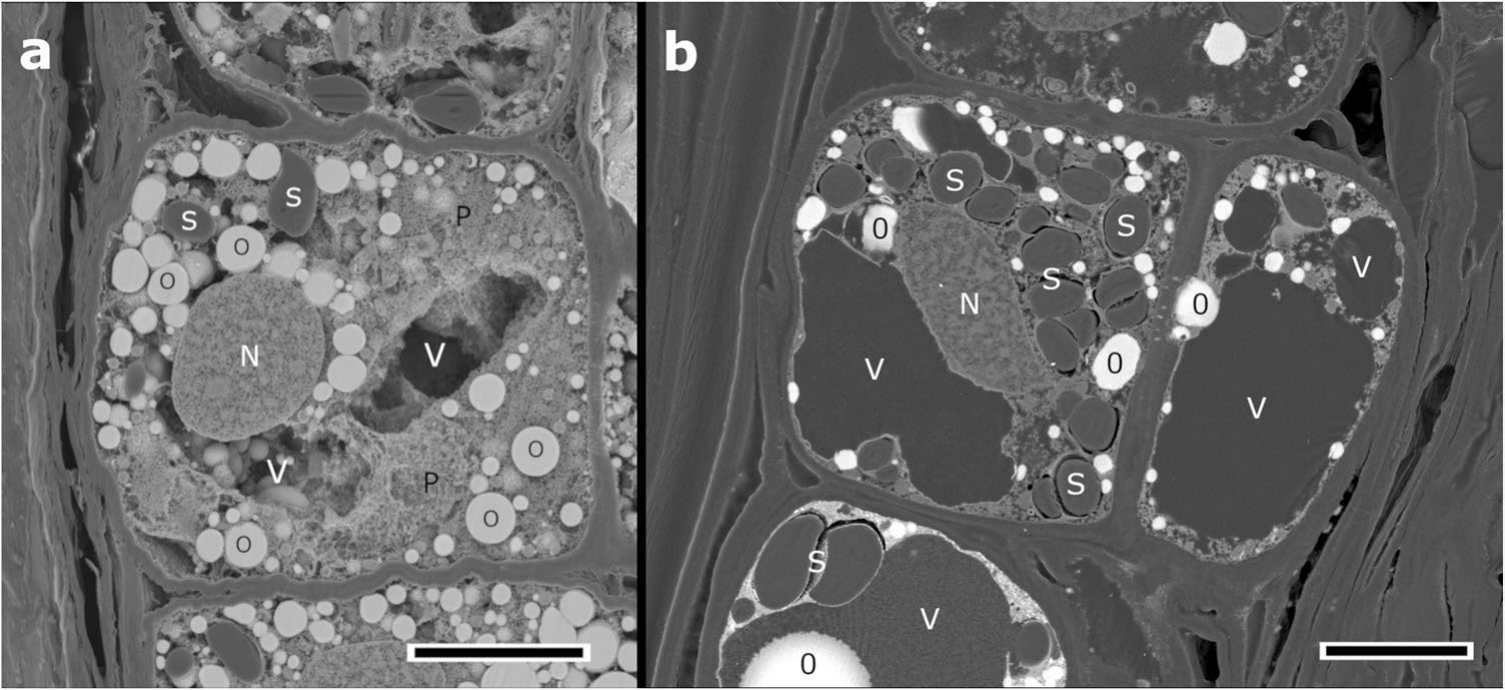
Radial sections of parenchyma cells in *Pinus densiflora* secondary phloem. (**a**) Radial section of broad argon ion beam milling (red-boxed area in Fig. 1a). Nuclei, protoplasmic 3D networks, and intracellular storage materials such as starch granules and oil bodies show good structural preservation without milling damage. The oil bodies exhibit a natural spherical shape, and vacuoles are void because epoxy resin embedding was not performed. (**b**) Radial microtome section. Starch granules were separated from the protoplasm by mechanical stress during the microtome cutting. The oil bodies were deformed by epoxy resin embedding, therefore presenting irregular shape, and the SEM image of the cell structure lost its depth information. N, nucleus; O, oil bodies; P, protoplasm; S, starch granules; V, vacuole. Scale bars = 20 µm.

Figure 2 shows the radial cross-sections of the parenchyma cells in the secondary phloem prepared by the BIB milling and microtome methods. The BIB milling method resulted in fine structural preservation of cell organelles and intracellular storage materials (Fig. 2a). The nuclei, starch granules, and oil bodies were observed in the BIB-milled cross-section without epoxy resin embedding. Additionally, because no epoxy resin was embedded, the oil bodies showed a natural spherical shape. The protoplasm was observed as 3D network structure that fixed intracellular storage material. The vacuoles appeared as voids owing to the leakage of the cell sap containing inorganic salts and water^34,35^ during the critical point drying process. In the microtome cross-section, starch granules were detached from the epoxy resin by mechanical stress during cutting (Fig. 2b). With epoxy resin embedding, the oil bodies were deformed to an amorphous sphere, and protoplasmic 3D networks and vacuoles were observed as planar images (Fig. 2b). In contrast, by not requiring epoxy resin embedding, the BIB milling method yielded 3D information of xylem tissues.

### Transverse sections of resin canals

**Figure 3:**
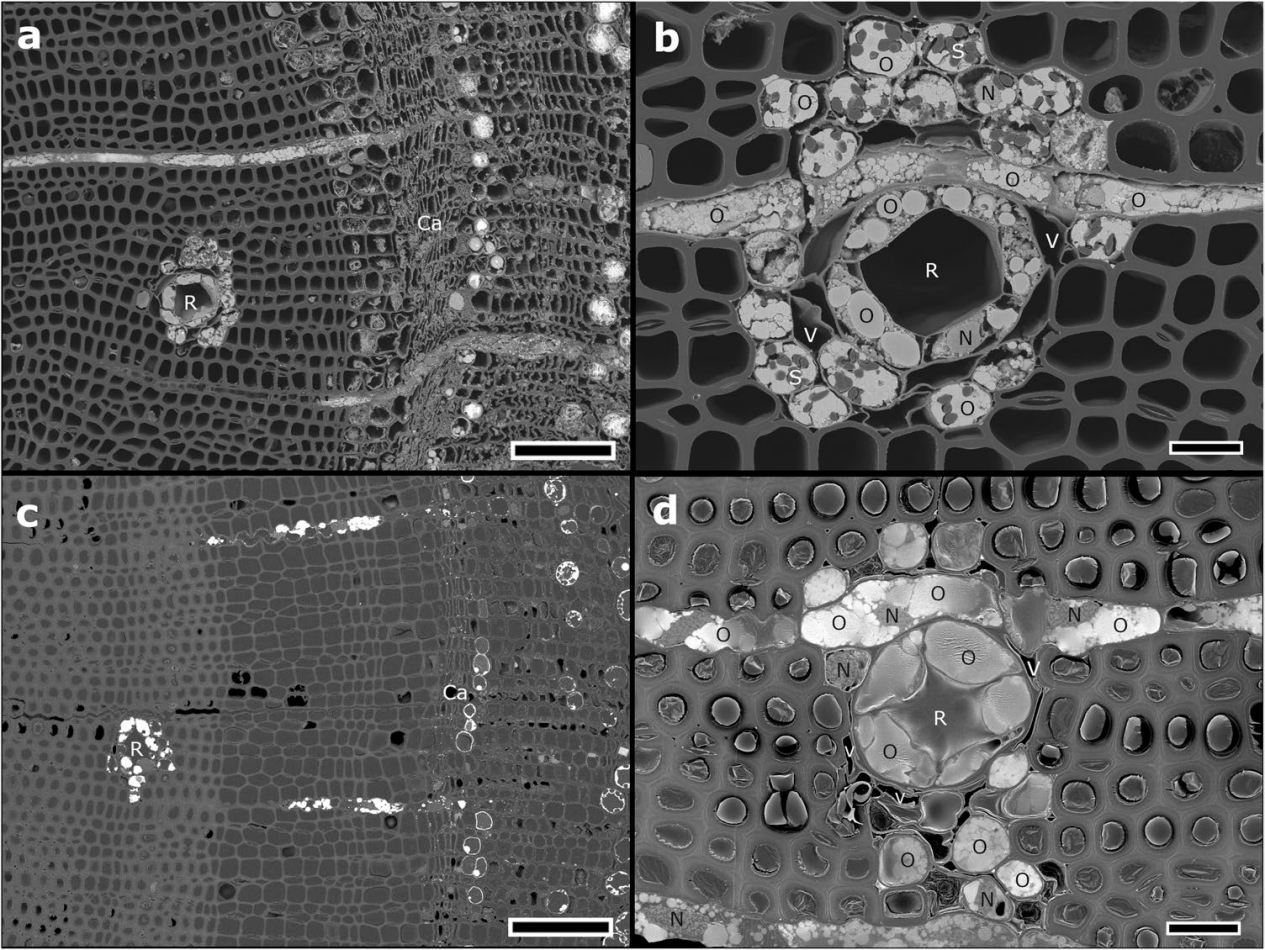
Transverse sections of *Pinus densiflora* resin canals and xylem parenchyma cells. (**a**,**b**) Broad argon ion beam (BIB)-milled cross-section. BIB milling created a broad and smooth cross-section from the phloem, cambium, and xylem, including the resin canal. (**b**) Parenchyma cells (epithelial, ray, and axial cells) contain many oil bodies (O) and have thinner cell walls than neighboring tracheids. No crushing of the thin cell walls or detachment of starch granules is observed in the BIB-milled cross-section. (**c**,**d**) Microtome cross-sections. The oil bodies are deformed into blurred outlines due to epoxy resin embedding. The epoxy resin is detached from the tracheids due to pine resin bleeding. Scanning electron microscopy images of intracellular storage materials are obscured by resin infiltration into epoxy resin. The resin canal and void spaces enclosed by thin cell walls are crushed by mechanical stress. Ca, cambium; N, nucleus; O, oil bodies; R, resin canal; S, starch granules; V, void space. Scale bars = 100 µm (**a** and **c**) and 20 µm (**b** and** d**).

Figure 3 shows transverse cross-sections of resin canals and parenchyma cells (epithelial, ray, and axial cells) prepared using the BIB milling and microtome methods. In the BIB-milled cross-section prepared without mechanical stress, we observed a broad area encompassing the phloem, cambium, and xylem, including resin canals (Fig. 3a). The cellular contents were readily identifiable because no epoxy resin embedding was performed (Fig. 3a). Further magnification of the resin duct revealed that the thin walls of parenchyma cells were not squashed and the inside of cells was filled with many spherical oil bodies and starch granules (Fig. 3b). The resin canal and void spaces enclosed within the thin cell walls retained their structure (Fig. 3b). On the contrary, in the microtome cross-section, the resin canal and void spaces were crushed by mechanical stress, and oil bodies appeared as amorphous spheres due to epoxy resin embedding (Fig. 3d). Moreover, in the microtome cross-section, the epoxy resin had high contrast due to pine resin infiltration, and SEM images of intracellular storage materials were obscured (Fig. 3d). In addition, the epoxy resin deteriorated by pine resin bleeding was detached from the tracheids (Fig. 3c,d).

### Cross-sectional observation of the reaction wood

**Figure 4:**
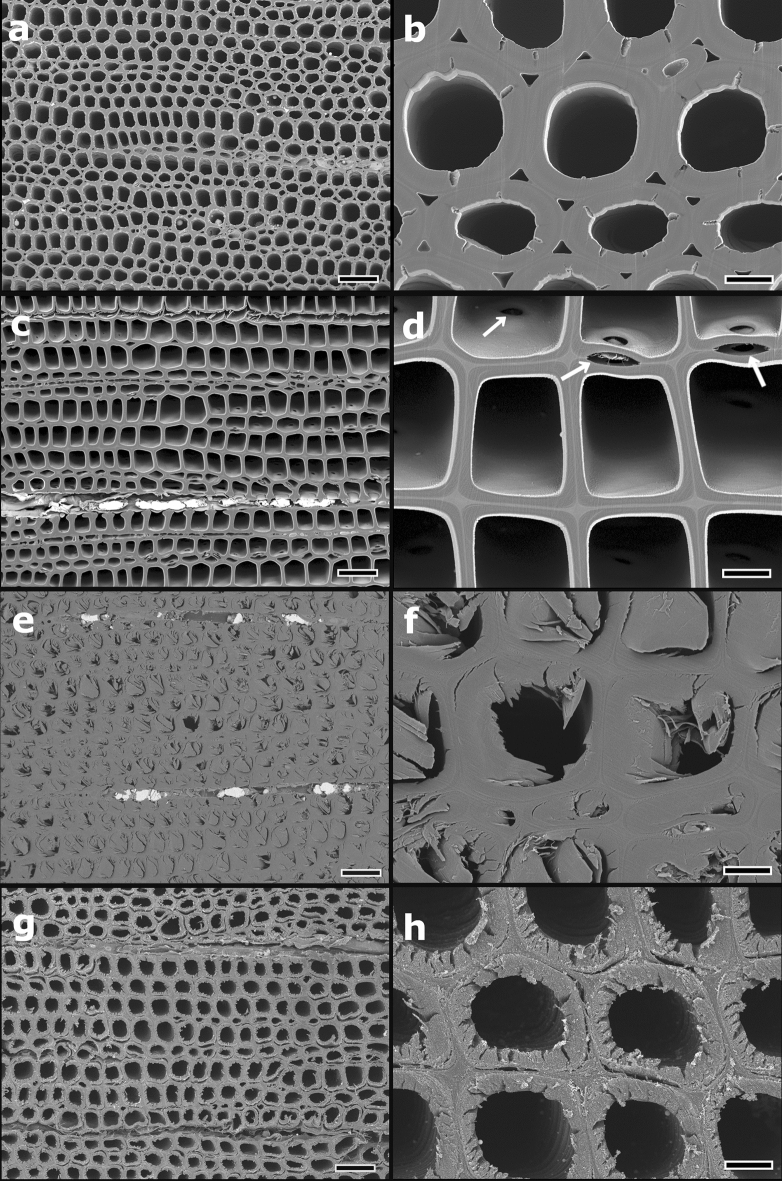
Transverse sections of tracheids in the compression wood and opposite wood of *Pinus densiflora*. (**a**,**b**) Broad argon ion beam (BIB)-milled cross-section of compression wood. The tracheids have a round shape with a thick cell wall. Many intercellular spaces can be observed among tracheids. No cutting damage, such as lightning bolt cracks or detachment between the S_1_ and S_2_ layers of the secondary wall, as observed in microtome cross-sections, are present in the BIB-milled section. (**c**,**d**) BIB-milled cross-section of opposite wood (non-reaction wood). Tracheids have rectangular or hexagonal shapes with relatively thin cell walls. Arrows indicate bordered pits. (**e**,**f**) Microtome cross-section of compression wood. The structures of cell walls and helical cavities are extended in the cutting direction. (**g**,**h**) Razor blade-cut cross-section of compression wood. Several cracks are generated in the S_2_ layer of the secondary wall (**e**,**h**). The S_2_ layers are delaminated from the S_1_ layers due to cutting stress. Scale bars = 50 µm (**a**, **c**, **e**, **g**) or 10 µm (**b**, **d**, **f**, **h**).

**Figure 5:**
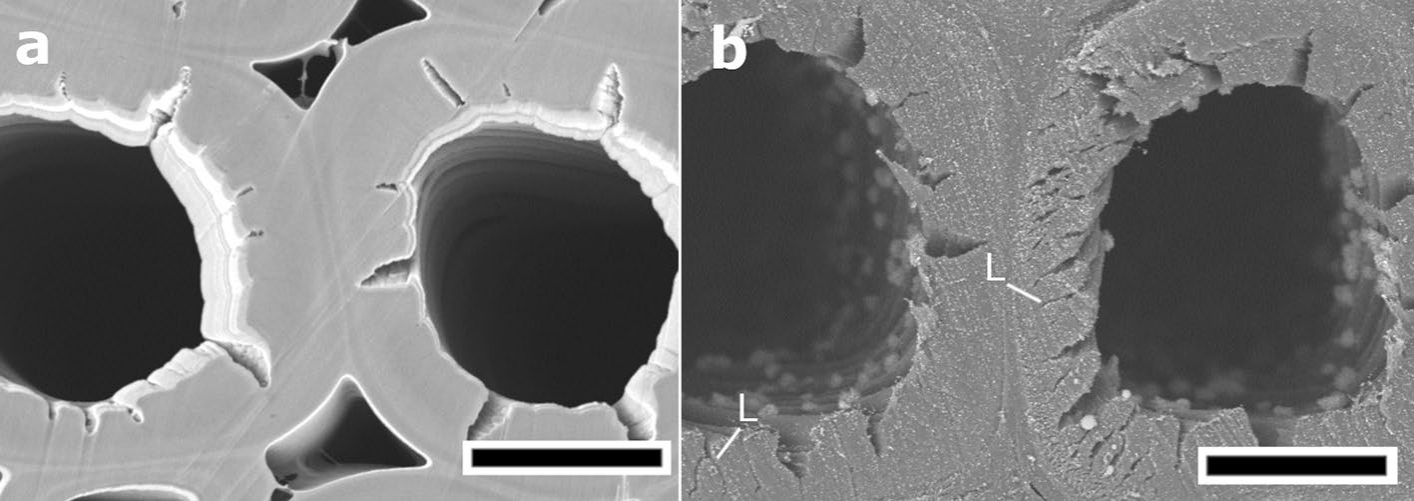
High-magnification image of transverse sections of *Pinus densiflora* compression wood. (**a**) Broad argon ion beam (BIB)-milled cross-section of compression wood. Cracks originating from the branched helical cavities are observed in the inner S_2_ layers of the secondary wall. (**b**) Razor blade-cut cross-section of compression wood. The lightning bolt-shaped cracks in the S_2_ layers are due to cutting stress. L, lightning bolt shape cracks. Scale bars = 10 µm.

Figures 4 and 5 show the transverse cross-sections of tracheids in compression wood and opposite wood (non-reaction wood) of *P. densiflora*. Opposite wood is defined as the xylem located opposite to the reaction wood formed at the leaning trunk^36^.

In the BIB-milled cross-section, compression wood tracheids exhibited a rounded shape with a thick cell wall, helical cavities, and many intercellular spaces (Figs. 4a,b, 5a). The highly lignified S_2_ layers showed a smooth cutting plane, no lightning bolt cracks or detachment between the S_1_ and S_2_ layers were observed (Figs. 4b, 5a). Tracheids of the opposite wood showed thin rectangular or hexagonal cell walls. Moreover, there were no intercellular spaces between individual tracheids (Fig. 4c,d). In the microtome cross-section of the compression wood, the structure was extended in the cutting direction; as a result, crushed helical cavities were exposed on the surface of the cutting section (Fig. 4e,f). In the razor blade-cut cross-section of compression wood, many cracks were generated in the secondary wall S_2_ layers, and these were detached from the outermost layer (S_1_ layer) of the secondary wall due to cutting (Figs. 4g,h, 5b).

**Figure 6:**
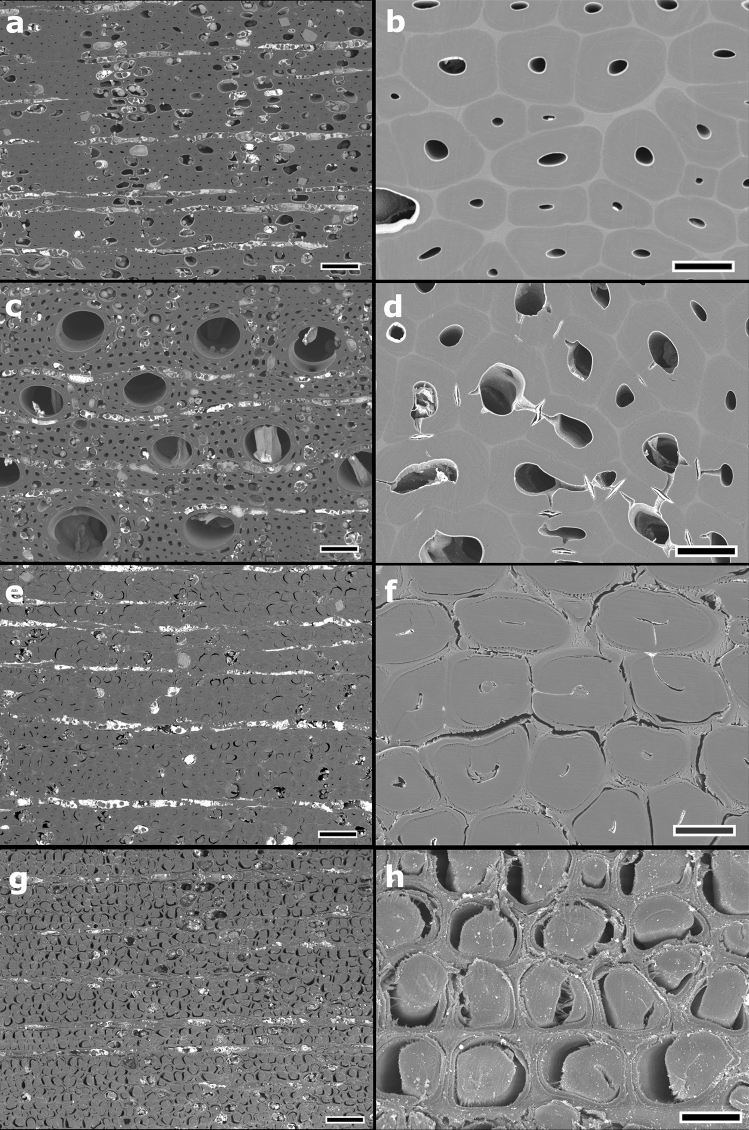
Transverse sections of tension wood and opposite wood of *Quercus phillyraeoides*. (**a**,**b**) Broad argon ion beam (BIB)-milled cross-section of tension wood. The cell lumens of wood fibers are reduced in size compared to those of opposite wood because of the gelatinous layer (G-layer) development. G-layers easily damaged by cutting stress were not observed as cutting artifacts over a wide range. (**c**,**d**) BIB-milled cross-section of opposite wood (non-reaction wood). Wood fibers have relatively large lumens and bordered pits. (**e**,**f**) Microtome cross-sections of tension wood. The G-layer and lumen are distorted due to mechanical stress. Detachment of the G-layer and numerous cracks are observed in a large area of the cross-section. (**g**,**h**) Razor blade-cut cross-section of tension wood. The G-layers are completely detached from the secondary walls. Scale bars = 50 µm (**a**, **c**, **e**, **g**) or 10 µm (**b**, **d**, **f**, **h**).

Figure 6 shows the transverse cross-sections of *Q. phillyraeoides* tension wood. In the gelatinous fibers of *Q. phillyraeoides*, BIB milling originated smooth and broad cutting surfaces (Fig. 6a,b). The cell lumens of gelatinous fibers were smaller in the tension wood than that in the non-reaction (opposite) wood (Fig. 6a–d). The elliptical-shaped G-layer showing detachment due to cutting-induced damage was not observed in the BIB-milled cross-sections (Fig. 6b). In contrast, in the microtome cross-section of gelatinous fibers, mechanical stress pressed and detached G-layers from the secondary walls (Fig. 6f), and cell wall shrinkage was extensive (Fig. 6e). In the razor blade-cut tension wood cross-section, mechanical stress detached most of the G-layers from the normal secondary walls (Fig. 6g,h).

## Discussion

To the best of our knowledge, this is the first study using BIB milling to prepare cross-sections of secondary tissues in woody plants without epoxy resin embedding. In addition, when combined with BIB milling, SEM provided accurate structural observations of secondary xylem cells because mechanical stress was not applied during the milling process. Controlling sample heating during BIB irradiation is essential in the biological sample milling process. Therefore, we mounted the BIB milling sample on a thin copper foil to prevent the effect of ion beam heating (Fig. 1b). The thin copper foil was attached to the shielding plate of the BIB milling system, transferring heat from the sample to the shielding plate. Using this procedure, the sample temperature during BIB irradiation can be suppressed to approximately 40 °C at an accelerating voltage of 4 kV^37^.

The parenchyma cells, which secrete resin, have fragile, thin walls that are prone to damage by mechanical wounding. In addition, granules of intracellular storage starch were detached from the microtome cross-section due to mechanical stress and oil bodies were irregularly shaped due to epoxy resin embedding (Fig. 2b). However, in the BIB-milled cross-section, the oil bodies showed a natural spherical shape, and detachment of the starch granules did not occur (Fig. 2a). Therefore, the BIB milling method, which does not require epoxy resin embedding and is based on sputtering, has the advantage of yielding more native structural information on intracellular storage materials.

In the microtome-section, tracheids of compression wood exhibited cracks originating from branched helical cavities in the middle layer (S_2_ layer) of the secondary wall, showing a lightning bolt shape due to mechanical damage^38^. In addition, detachment frequently occurred between the S_1_ and S_2_ layers due to mechanical stress. Tension wood is generally characterized by gelatinous fibers with a thick inner G-layer^39^. In the tension wood G-layer, structural damage and artifacts occur due to mechanical sectioning. The G-layer detachment from normal secondary walls observed in microtome sections is caused by the mechanical cutting of the transverse face^40–43^. Contrastingly, the BIB-milling method can be used to prepare large sections without cutting artifacts and distortion in fragile compression wood and hardwoods with high density and stiff xylem cells, such as *Q. phillyraeoides* (Figs. 4b, 5a, 6b,d). Therefore, the BIB milling method allows accurately visualizing compression wood and tension wood structures without applying mechanical stress. However, some artifacts occurring in living tissues during the pretreatment process of BIB-milling are unavoidable. These include cell membrane damage during the chemical fixation process^44^ and tissue shrinkage during critical point drying^45–47^.

In addition to the microtome method, freeze-fracture is used as a cross-section processing method for SEM observations of plant samples^48,49^. In the freeze-fracture method, the fracture plane tends to pass through the center of lipid bilayers in cell membranes, exposing the extracellular face (EF) and protoplasmic face (PF)^50^. The rosette cellulose synthesis complex is observed as six particles in freeze-fracture replicates of the protoplasmic face^51,52^. In SEM observations, freeze-fractured samples are generally observed using secondary electrons providing information on the morphology and surface topography^48,49^. In contrast, BIB milled samples are suited for imaging with backscattered electrons providing information on material composition and density because the ion etched surfaces are extremely smooth. In the present study, all SEM images were obtained using a back-scattered electron detector. Therefore, the BIB milling SEM images closely resemble TEM images.

The results presented above indicate there are many advantages of using the BIB milling technique followed by SEM for analyzing the fine structure of a mixture of hard and soft secondary xylem cells. We therefore believe that this method is significant and useful for studies in plant science.

## Methods

The plants *P. densiflora* and *Q. phillyraeoides* were obtained from the Tokyo University of Agriculture and Technology, Tokyo, Japan. First, branches were cut with a sharp single-edged razor blade into small pieces of two square millimeters and immersed in 2.5% glutaraldehyde in sodium phosphate buffer (0.1 M) (pH 7.4) overnight at 4 °C. Thereafter, samples were rinsed five times with sodium phosphate buffer (0.1 M) (pH 7.4) and post-fixed in osmium tetroxide (1.0%) and sodium phosphate buffer (0.1 M) (pH 7.4) for 2 h at 25 °C. Samples were then rinsed thrice in sodium phosphate buffer (0.1 M) (pH 7.4). Finally, samples were dehydrated in a graded series of ethanol for 15 min at each step. The cutting planes for the BIB milling system and ultra-microtome were coated with osmium (1.5 nm) using an osmium plasma coater (HPC-20, Shinkuu, Mito, Japan). The coated samples were observed under a scanning electron microscope (JSM-7900F, JEOL, Akishima, Japan) at an accelerating voltage of 5 kV. Images were taken using a back-scattered electron detector. Finally, species were identified using botanical materials, and samples were stored in the laboratory.

### BIB cross-sectioning of secondary woody tissues and reaction wood

Samples were immersed in 100% ethanol and critical point dried in a critical point dryer (SYSGLCP-8, Sanyu-Gijutsu, Akiruno, Japan) with a purging flow rate of 1.0 L/min at 40 °C. The dried samples were mounted on a copper foil (99.95% purity, 0.1-mm-thick, Nilaco, Tokyo, Japan) with a two-component epoxy resin adhesive (Quick 5, Konishi, Osaka, Japan) and subsequently attached to the BIB milling system (IB-19520, JEOL). BIB milling was performed at an accelerating voltage of 4 kV and milling time of 8 h.

### Ultra-microtome cross-sectioning of secondary woody tissues

The dehydrated samples were immersed in a 1:1 mixture of Spurr epoxy resin (Polysciences, Warrington, PA, USA), dehydrated using ethanol, and rotated at 4 rpm and 25 °C for 2 h on a rotator (TAAB Laboratories Equipment, Aldermaston, UK). The samples were then immersed in 100% Spurr epoxy resin and rotated overnight. Finally, samples were embedded in 100% Spurr epoxy resin and polymerized at 70 °C for 8 h. The embedded samples were cut with an ultra-microtome (UltraCut UCT Type 706200, Leica, Munich, Germany) in 90 nm steps with a 45° diamond knife (Diatome, Helmstrasse, Switzerland). Resin blocks with a mirror finish were used for SEM observation without thin sectioning.

### Ultra-microtome cross-sectioning of reaction wood

The critically point dried samples (not epoxy embedded) were cut with an ultra-microtome (UltraCut UCT Type 706200, Leica) in 90 nm steps with a 45° diamond knife (Diatome).

### Cutting of reaction wood

The glutaraldehyde-fixed wet samples were cut with a single-edged razor blade before immersion in an osmium tetroxide fixative.

### Statement of compliance

Experimental research on plants and collection of plant materials were performed in accordance with relevant institutional, national, and international guidelines and legislation.

## Data Availability

All data generated or analyzed during this study are included in this article.
